# Choroid plexus volume variation in patients with gliomas, glioneuronal and neuronal tumors: Associations with tumor characteristics

**DOI:** 10.1371/journal.pone.0357498

**Published:** 2026-09-15

**Authors:** Jia Sun, Minghao Wu, Junjie Li, Jinyuan Weng, Renlong Zhang, Yuanbin Zhao, Xiaoqin Zhu, Mengzhao Li, Yunyun Duan, Cong Zhang, Dongli Shi

**Affiliations:** 1 Department of Radiology, Peking University Third Hospital, Beijing, China; 2 Department of Radiology, Beijing Tiantan Hospital, Capital Medical University, Beijing, China; 3 Key Laboratory of Carcinogenesis and Translational Research (Ministry of Education/Beijing), Department of Interventional Therapy, Peking University Cancer Hospital, Beijing, China; 4 Department of Clinical and Technical Support, Philips Healthcare, Shanghai, China; 5 Institute of Research and Clinical Innovations, Neusoft Medical Systems Co., Ltd., Beijing, China; 6 Department of Radiology, Tianjin Key Laboratory of Functional Imaging, and State Key Laboratory of Experimental Hematology, Tianjin Medical University General Hospital, Tianjin, China; 7 Department of Internal Medicine II, Rongcheng County People’s Hospital, Xiongan New Area, Hebei, China; 8 Department of Neurology, the Second Hospital of Hebei Medical University, Shijiazhuang, Hebei, China; Università degli Studi di Napoli Federico II: Universita degli Studi di Napoli Federico II, ITALY

## Abstract

**Purpose:**

To investigate choroid plexus .   volume (CPV) alterations in patients with gliomas and glioneuronal and neuronal tumors (GNTs) and to evaluate their associations with tumor characteristics.

**Methods:**

In this retrospective observational cohort study, 811 patients with histologically confirmed brain tumors (674 gliomas and 137 GNTs) and 744 controls were included. All participants underwent 3.0 T MRI with 3D T1-weighted imaging, and patient scans were acquired preoperatively. The choroid plexus was automatically segmented on 3D T1-weighted images using a 3D nnU-Net–based deep learning algorithm with expert quality control. CPV was normalized to total intracranial volume (CPV/TIV). Lateral ventricular volume (LVV) was quantified using FreeSurfer and included as a covariate. Group comparisons were performed using Mann–Whitney U and Kruskal–Wallis tests after case-control matching. Multivariable linear regression analyses were conducted to assess independent associations while adjusting for age, sex, and LVV, with additional sensitivity analyses incorporating scanner type.

**Results:**

Compared with supratentorial tumors and controls, infratentorial tumors showed significantly larger CPV (both *P* < 0.001). Tumor grade was not independently associated with CPV after adjustment among patients. Pediatric-type diffuse high-grade gliomas and GNTs exhibited larger CPV than adult-type diffuse gliomas (*P* < 0.01). In exploratory molecular analyses of adult-type diffuse gliomas, + 7/ − 10 status was associated with lower CPV (*β* = −0.127, *P* = 0.009).

**Conclusion:**

CPV alterations in brain tumors are primarily associated with tumor location and histological classification rather than tumor grade. An exploratory association between +7/ − 10 status and lower CPV was also observed, although this finding requires further validation.

## Introduction

Gliomas and glioneuronal and neuronal tumors (GNTs) represent the most prevalent primary tumors of the central nervous system (CNS). The fifth edition of the WHO Classification of Tumors of the Central Nervous System (WHO CNS5) [1] classifies gliomas and GNTs into distinct families and four grades based on integrated histopathological and molecular features.

Recent studies have shown that gliomas induce immunosuppression [2–4] and elicit an inflammatory response within the tumor microenvironment [5–7], thereby promoting tumor progression and limiting therapeutic efficacy. Additionally, inflammation associated with gliomas can compromise the integrity of the blood–brain barrier [5,8], disrupting CNS homeostasis and immune regulation.

Meanwhile, the choroid plexus (ChP), situated in the lateral, third, and fourth ventricles, plays a central role in CNS immune surveillance and inflammatory responses [9–11]. As a major component of the blood-cerebrospinal fluid (CSF) barrier, the ChP regulates immune cell trafficking between the bloodstream and the brain, maintaining homeostasis and responding to systemic or local inflammation [12,13]. Although ChP alterations are well-documented in neurodegenerative and neuroimmune diseases such as Alzheimer’s disease and multiple sclerosis [14–16], its specific alterations in gliomas and GNTs remain largely unknown.

Building on these observations, this study aimed to characterize CPV alterations in patients with gliomas and GNTs and to evaluate their associations with anatomical location, histological type, grade, and molecular biomarkers, thereby exploring the potential of CPV as a non-invasive imaging marker in brain tumors.

## Materials and methods

### Study participants

Clinical and imaging data used in this study were accessed for research purposes between 01/03/2023 and 30/04/2024. We reviewed medical records and preoperative MRI data of 1,200 patients diagnosed with gliomas or GNTs who underwent MRI at Beijing Tiantan Hospital between May 2016 and September 2022. After applying the exclusion criteria, 811 patients were included (674 gliomas and 137 GNTs). The study was conducted in accordance with the Declaration of Helsinki and was approved by the Institutional Review Board of Beijing Tiantan Hospital (Approval No. KY2022-078-04). The requirement for written informed consent was waived due to the retrospective nature of the study.

Inclusion criteria were: histopathological and molecular confirmation of glioma or GNTs according to the WHO CNS5 classification, and availability of high-quality 3D T1-weighted MRI with clear ChP visualization and sufficient clinical and pathological data. Patients were excluded if they had substantial tumor-related compression of the lateral ventricles or ChP that interfered with segmentation, recurrent tumors, prior radiotherapy or chemotherapy, or neurological disorders potentially affecting ChP morphology.

**Fig 1:**
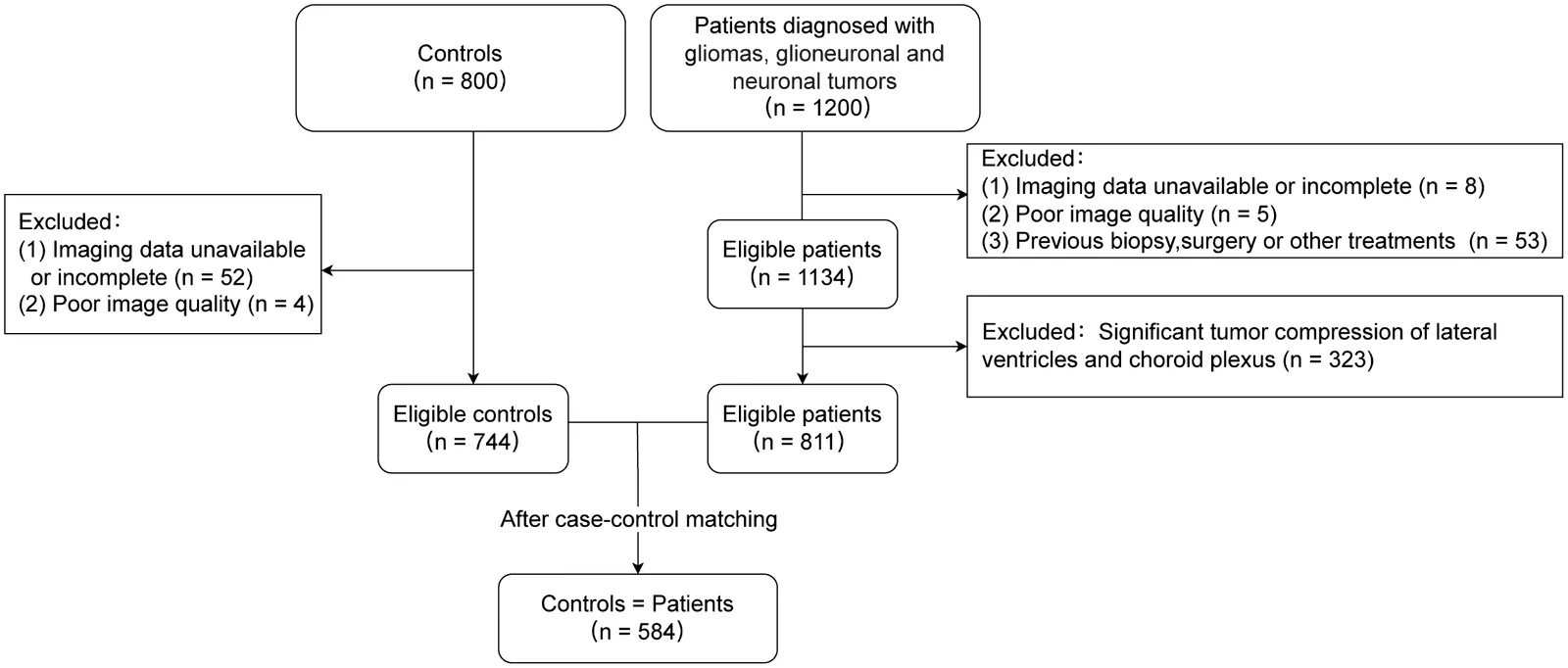
Flowchart showing the study inclusion and exclusion process.

MRI data for a total of 800 potential controls were retrospectively obtained from two sources. After excluding 52 cases with incomplete imaging data and 4 with poor image quality, 744 controls were included in the final control cohort, comprising 542 healthy individuals who had previously undergone brain MRI at Beijing Tiantan Hospital and 202 healthy individuals from the publicly available Developing Chinese Color Nest Project (devCCNP, 2013–2022) dataset, which was collected for normative brain development studies in healthy individuals [17]. None of the controls had a history of central nervous system disorders, and their MRIs were normal or showed only mild age-related white-matter changes (Fazekas = 1). A flowchart of participant selection is shown in Fig 1.

### Clinical and pathological features

Clinical and pathological data were meticulously extracted from electronic medical records, covering demographic details (e.g., age, sex) and key pathological information. To account for age-related effects, participants were categorized into four age groups: pediatric (<18 years), young adults (18–44 years), middle-aged (45–59 years), and elderly (≥60 years).

Patients were classified into four groups based on WHO CNS5 classification: 1) Adult-type diffuse gliomas (Astrocytoma, Oligodendroglioma, Glioblastoma); 2) Pediatric-type diffuse high-grade gliomas (Diffuse midline glioma); 3) Circumscribed astrocytic gliomas (Pilocytic astrocytoma, Pleomorphic xanthoastrocytoma); 4) GNTs (a diverse group with neuronal differentiation). The detailed histological composition of the GNT cohort is provided in S1 Table in S1 File.

Additionally, tumor location (supratentorial or infratentorial) and grade (1–4) were recorded. Molecular biomarkers included mutations of isocitrate dehydrogenase 1 and 2 (IDH), codeletion of chromosomal arms 1p/19q, telomerase reverse transcriptase (TERT) promoter mutations, O^6^-methylguanine-DNA methyltransferase (MGMT) promoter methylation, alpha-thalassemia/mental retardation X-linked syndrome (ATRX) gene mutations, epidermal growth factor receptor (EGFR) amplification, combined gain of chromosome 7 and loss of chromosome 10 (+7/ − 10), and loss of cyclin-dependent kinase inhibitor 2A/B (CDKN2A/B) [18]. Pathology data were obtained from tumor tissue testing using immunohistochemistry, next-generation sequencing, and pyrosequencing. Only patients with definitive molecular testing results were included in the MGMT and TERT analyses. For other biomarkers, a combination of immunohistochemistry and sequencing results was used.

### MRI data acquisition

All MRI scans were performed on various 3.0 T MR scanners using a 3D T1-weighted sequence protocol. A neuroradiologist (J. Sun, with 6 years of experience in neuroradiology) reviewed all scans to ensure adequate image quality and confirmed that all patient scans were acquired preoperatively. The main acquisition parameters were as follows: flip angle (FA) = 8°-12°; for GE and Philips scanners, repetition time/echo time (TR/TE) = 6.4–11.2 ms/ 3.0–5.1 ms; for Siemens scanners, TR/TE = 1560–2600 ms/ 1.7–3.0 ms; inversion time (TI) = 450–880 ms; spatial resolution = 1 × 1 × 1 mm³; matrix size = 220–256 × 218–256; and slice numbers = 170–196. Detailed MRI protocol parameters for each scanner are provided in S2 Table in S1 File.

### Measurement of CPV, total intracranial volume and lateral ventricular volume

**Fig 2:**
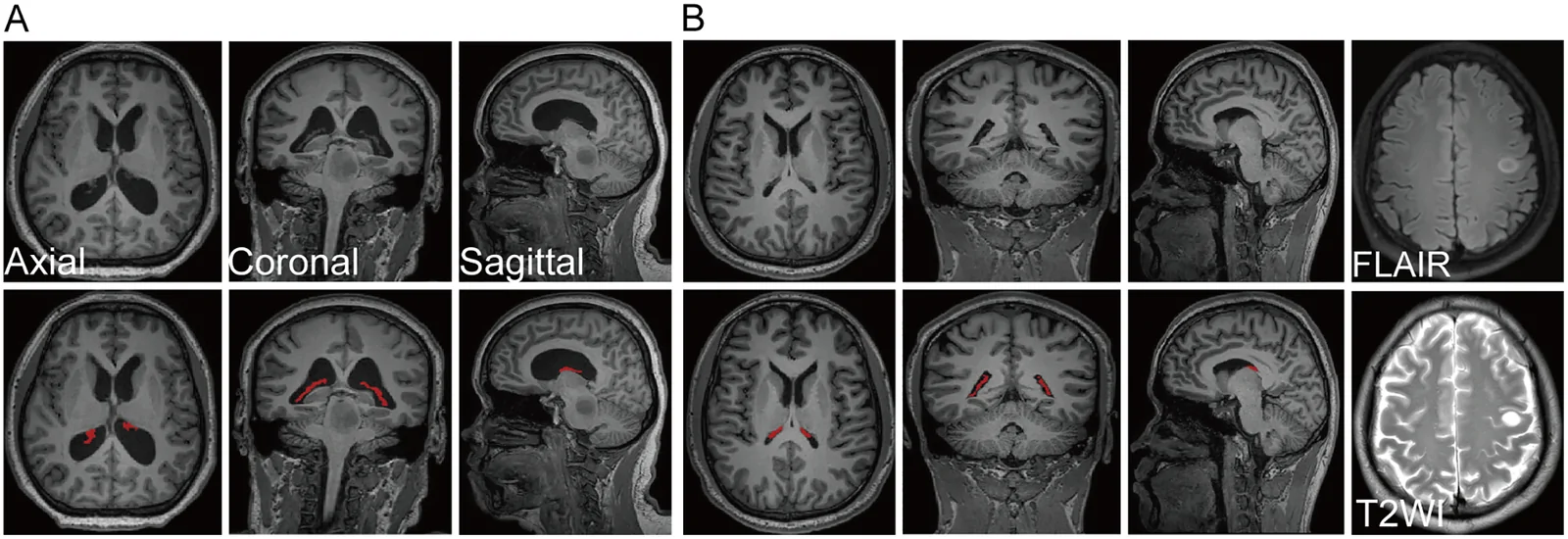
Segmentation of the choroid plexus (ChP) on 3.0 T brain MRI. Representative examples of the ChP (red) in patients with tumors at different locations. (A) A 34-year-old patient with a diffuse midline glioma located in the brainstem. (B) A 34-year-old patient with an astrocytoma located in the supratentorial frontal lobe. The ChP volume is notably larger in the patient with the infratentorial tumor compared with the patient with the supratentorial tumor.

The ChP within the lateral ventricles was segmented using an internally developed deep learning algorithm based on the 3D nnU-Net framework [19]. The model was trained using 50 manually annotated cases and was additionally evaluated in 30 cases. The model achieved a mean Dice similarity coefficient of 0.909 ± 0.044. Representative ChP segmentation examples are shown in Fig 2, and details of model training and validation are provided in the Supporting Information.

The total intracranial volume (TIV) was determined using the HD-BET algorithm [20], a rigorously validated method based on artificial neural networks. HD-BET was developed using MRI data obtained from a large multicenter clinical trial involving adult brain tumor patients from 37 institutions across Europe. The dataset encompassed a wide range of MR hardware and acquisition parameters, as well as various pathologies and treatment-induced tissue alterations.

Lateral ventricular volume (LVV), including both left and right ventricles together with their temporal horns, was quantified using FreeSurfer (version 7.3.2). These measurements were incorporated into subsequent analyses to account for potential effects of ventricular enlargement or hydrocephalus on CPV.

All automated segmentation results for the ChP, TIV, and LVV were visually reviewed by two experienced neuroimaging researchers (J. Sun and Y. Duan). Manual correction was required for ChP segmentation in 22 of 1,555 cases and for LVV segmentation in 7 cases. No manual correction was required for TIV segmentation. Manual corrections were performed by J. Sun using ITK-SNAP (version 4.0.1), while blinded to clinical and pathological information.

### Statistical analysis

Statistical analyses were performed using SPSS (version 29.0). Similar to previously published studies [16,21], CPV was normalized to TIV by calculating the CPV/TIV ratio to minimize individual differences in head size. For ease of interpretation, CPV/TIV values were expressed in units of 10^−3^ in descriptive and regression analyses.

A 1:1 case-control matching procedure was performed based on age and sex. Sex was matched exactly, and an age difference of up to 2 years was permitted. After matching, the Mann–Whitney U test was used for two-group comparisons, and the Kruskal–Wallis test was employed for comparisons among multiple groups. Bonferroni correction was applied for multiple comparisons.

Subsequently, multivariable linear regression analyses were conducted in the full cohort without matching to evaluate the associations between CPV/TIV and tumor-related variables. CPV/TIV was specified as the dependent variable, and age, sex, and LVV were included as covariates in all models. Tumor location was evaluated in Model 1; tumor type and tumor grade were evaluated in Models 2 and 3, respectively, with tumor location additionally included in Models 2 and 3. Categorical variables with more than two levels were entered into the regression models using dummy coding. Covariate-adjusted pairwise comparisons were obtained by refitting the corresponding regression models using different reference categories. Multicollinearity was assessed using variance inflation factors (VIFs), and no problematic multicollinearity was identified. To assess the potential influence of scanner-related heterogeneity, additional sensitivity analyses were performed by incorporating scanner type (five categories) as a categorical covariate.

For molecular biomarker analyses restricted to adult-type diffuse gliomas, separate multivariable linear regression models were performed for each biomarker, adjusting for age, sex, and LVV, with scanner type additionally included in sensitivity analyses. For the + 7/ − 10 analysis, further sensitivity analyses were performed with separate adjustment for tumor location, WHO grade, tumor subtype, and IDH status.

A two-sided *P* value < 0.05 was considered statistically significant.

## Results

### Baseline characteristics and comparisons between controls and patients

After applying exclusion criteria, this study analyzed data from 811 patients (674 with gliomas and 137 with GNTs), aged 1–74 years, and 744 controls, aged 6–79 years. Baseline characteristics are presented in Table 1.

**Table 1:** Demographic characteristics of all participants at baseline.

	Controls	Patients	Adult-type diffuse gliomas			Pediatric-type diffuse high-grade gliomas	Circumscribed astrocytic gliomas		Glioneuronal and neuronal tumors
	(n = 744)	(n = 811)	Astrocytoma (n = 176)	Oligodendroglioma (n = 162)	Glioblastoma (n = 99)	Diffuse midline glioma (n = 112)	Pilocytic astrocytoma (n = 107)	Pleomorphic xanthoastrocytoma (n = 18)	GNTs (n = 137)
Age (years)									
< 18	202 (27.2%)	211 (26.0%)	5 (2.8%)	1 (0.6%)	3 (3.0%)	86 (76.8%)	61 (57.0%)	8 (44.4%)	47 (34.3%)
18–44	165 (22.2%)	337 (41.6%)	112 (63.6%)	74 (45.7%)	24 (24.2%)	18 (16.1%)	32 (29.9%)	8 (44.4%)	69 (50.4%)
45–59	292 (39.2%)	188 (23.2%)	40 (22.7%)	74 (45.7%)	36 (36.4%)	8 (7.1%)	11 (10.3%)	2 (11.1%)	17 (12.4%)
≥ 60	85 (11.4%)	75 (9.2%)	19 (10.8%)	13 (8.0%)	36 (36.4%)	NA	3 (2.8%)	NA	4 (2.9%)
Sex									
Male	407 (54.7%)	420 (51.8%)	97 (55.1%)	90 (55.6%)	59 (59.6%)	53 (47.3%)	53 (49.5%)	11 (61.1%)	57 (41.6%)
Female	337 (45.3%)	391 (48.2%)	79 (44.9%)	72 (44.4%)	40 (40.4%)	59 (52.7%)	54 (50.5%)	7 (38.9%)	80 (58.4%)
Location									
Supratentorial	NA	568 (70.0%)	159 (90.3%)	162 (100%)	94 (94.9%)	5 (4.5%)	24 (22.4%)	18 (100%)	106 (77.4%)
Infratentorial	NA	243 (30.0%)	17 (9.7%)	NA	5 (5.1%)	107 (95.5%)	83 (77.6%)	NA	31 (22.6%)
Grade									
1	NA	241 (29.7%)	NA	NA	NA	NA	107 (100%)	NA	134 (97.8%)
2	NA	244 (30.1%)	121 (68.8%)	104 (64.2%)	NA	NA	NA	16 (88.9%)	3 (2.2%)
3	NA	101 (12.5%)	41 (23.3%)	58 (35.8%)	NA	NA	NA	2 (11.1%)	NA
4	NA	225 (27.7%)	14 (8.0%)	NA	99 (100%)	112 (100%)	NA	NA	NA

GNTs, glioneuronal and neuronal tumors; NA, not applicable.

**Fig 3:**
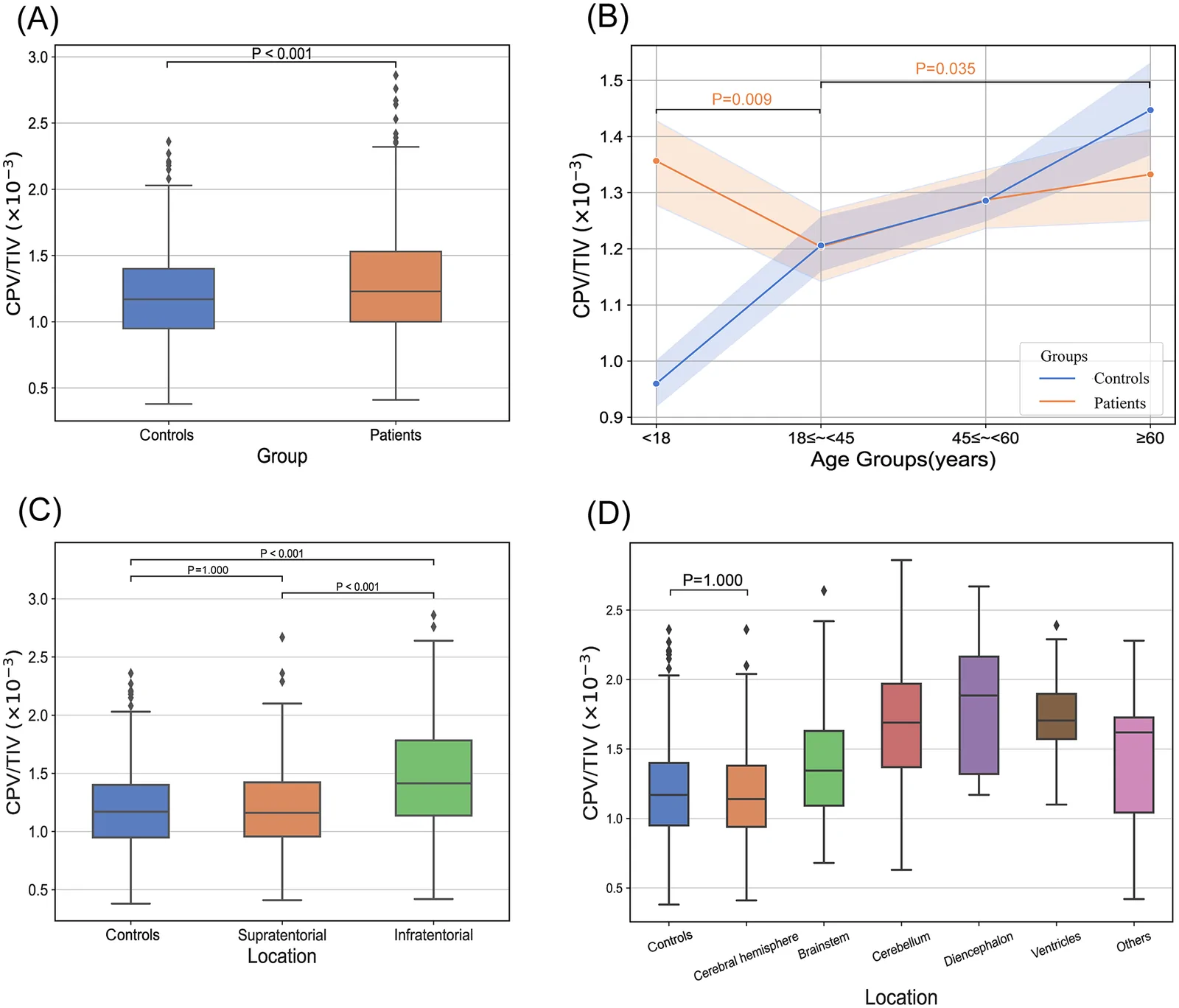
(A) Comparison of CPV/TIV between controls and patients after case-control matching. (B) Comparison of CPV/TIV across age groups. Significant differences were observed between pediatric and young adult patients (*P* = 0.009), and between elderly and young adult patients (*P* = 0.035). (C-D) Comparison of CPV/TIV across different tumor locations. Abbreviations: CPV, choroid plexus volume; TIV, total intracranial volume.

After matching, 584 pairs of controls and patients were successfully matched. Patients exhibited significantly larger CPV compared with controls (*P* < 0.001). This difference was consistent before and after matching (Fig 3A and S3 Table in S1 File).

To further examine age-related effects, CPV was compared across four age groups within each cohort. Among controls, CPV increased progressively with age (all pairwise *P* < 0.05). Among patients, significant differences were observed between pediatric and young adult groups (*P* = 0.009) and between elderly and young adult groups (*P* = 0.035) (Fig 3B and S4 Table in S1 File).

Inter-cohort comparisons within the same age group revealed that pediatric patients had significantly larger CPV than pediatric controls (*P* < 0.001), whereas elderly patients had slightly smaller CPV than elderly controls (*P* = 0.048). No significant differences were observed in the young adult or middle-aged groups (all *P* > 0.05; S5 Table in S1 File).

### Impact of tumor locations on CPV

CPV differed markedly according to tumor location. Patients with infratentorial tumors showed significantly larger CPV than both supratentorial tumor patients and controls (both *P* < 0.001; Fig 3C). No difference was detected between supratentorial tumors and controls (*P* = 1.000).

When patients were further categorized into six anatomical subregions—cerebral hemisphere, brainstem, cerebellum, diencephalon, ventricles, and other regions—CPV values in the brainstem, cerebellum, diencephalon, and ventricular tumor groups were all significantly higher than those in the cerebral hemisphere group and in controls (all Bonferroni-adjusted *P* < 0.05; Fig 3D and Table 2). No significant differences were observed for tumors in “other regions of the brain”.

**Table 2:** Comparison of CPV/TIV between controls and patients after matching.

	Number (%)	CPV/TIV [×10^-3^, median (IQR)]	Kruskal-Wallis H	
			*H*	*P*
Location				
Controls	584 (100%)	1.17 (0.95–1.40) ^a^	54.41	<0.001
Supratentorial	412 (70.5%)	1.16 (0.96–1.42) ^a^		
Infratentorial	172 (29.5%)	1.41 (1.13–1.78) ^b^		
Location				
Controls	584 (100%)	1.17 (0.95–1.40) ^a^	88.13	<0.001
Cerebral hemisphere	391 (67.0%)	1.14 (0.94–1.38) ^a^		
Brainstem	126 (21.6%)	1.34 (1.09–1.63) ^b^		
Cerebellum	37 (6.3%)	1.69 (1.36–2.04) ^b^		
Diencephalon	8 (1.4%)	1.88 (1.26–2.30) ^b^		
Ventricles	10 (1.7%)	1.70 (1.47–2.00) ^b^		
Others	12 (2.1%)	1.62 (0.95–1.77) ^ab^		
Grade				
Controls	584 (100%)	1.17 (0.95–1.40) ^a^	27.25	<0.001
1	139 (23.8%)	1.34 (0.96–1.69) ^b^		
2	177 (30.3%)	1.19 (1.00–1.40) ^ab^		
3	77 (13.2%)	1.16 (0.92–1.41) ^ac^		
4	191 (32.7%)	1.25 (1.05–1.59) ^bc^		
Classification				
Controls	584 (100%)	1.17 (0.95–1.40) ^a^	49.71	<0.001
Adult-type diffuse gliomas	335 (57.4%)	1.18 (0.99–1.44) ^a^		
Pediatric-type diffuse high-grade gliomas	93 (15.9%)	1.35 (1.08–1.74) ^b^		
Circumscribed astrocytic gliomas	78 (13.4%)	1.49 (1.15–1.86) ^b^		
Glioneuronal and neuronal tumors	78 (13.4%)	1.11 (0.90–1.42) ^a^		

Groups sharing a common letter in the CPV/TIV column do not significantly differ, groups with different letters show significant differences. Significance values have been adjusted using the Bonferroni correction for multiple tests. Abbreviations: CPV, choroid plexus volume; TIV, total intracranial volume.

### Comparison of CPV across tumor grades and types

**Fig 4:**
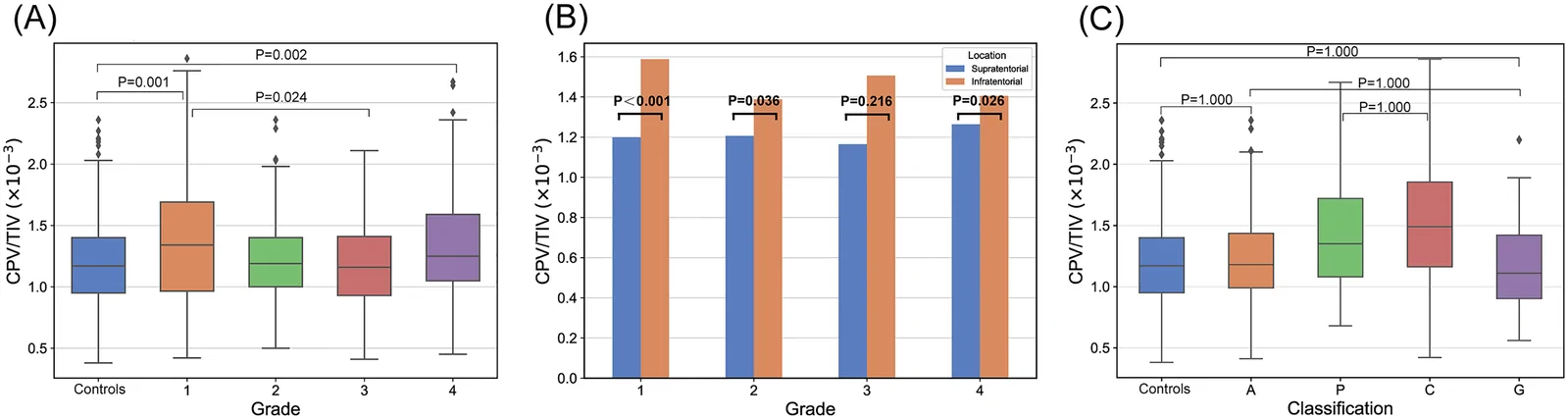
(A) Comparison of CPV/TIV across different grades. (B) Comparison of CPV/TIV across grades and locations. (C) Comparison of CPV/TIV across different tumor types. Abbreviations: A, Adult-type diffuse gliomas; P, Pediatric-type diffuse high-grade gliomas; C, Circumscribed astrocytic gliomas; G, Glioneuronal and neuronal tumors; CPV, choroid plexus volume; TIV, total intracranial volume.

After matching, CPV comparisons were performed across tumor grades and histological types. For tumor grades, CPV was significantly larger in patients with Grade 1 and Grade 4 tumors than in controls (*P* = 0.001 and *P* = 0.002, respectively) and was also larger in the Grade 1 group than in the Grade 3 group (*P* = 0.024), while no significant differences were observed among the remaining groups (all *P* > 0.05; Fig 4A and Table 2).

When patients were grouped by both grade and location, CPV was consistently larger in patients with infratentorial tumors than in those with supratentorial tumors for Grades 1, 2, and 4 (all *P* < 0.05), but not for Grade 3 (*P* = 0.216; Fig 4B and S6 Table in S1 File). The lack of significance in Grade 3 tumors was likely due to the small number of infratentorial cases in this subgroup (n = 3), which limited statistical power.

Separate analyses revealed no significant CPV differences among grades within either the supratentorial or infratentorial groups (both *P* > 0.05).

Across histological types, patients with pediatric-type diffuse high-grade gliomas and circumscribed astrocytic gliomas exhibited significantly larger CPV than those with adult-type diffuse gliomas or GNTs (all *P* < 0.01; Fig 4C and Table 2). CPV in adult-type diffuse gliomas was comparable to that in controls and GNTs (all *P* = 1.000). To further explore the differences in CPV within each tumor type, we performed case-control matching separately between controls and patients across four tumor categories. Detailed results are presented in the supplementary materials.

### Multivariable analysis of factors associated with CPV

Multivariable linear regression analyses were then performed to examine the independent associations between CPV and tumor characteristics.

#### Regression analysis of the effect of anatomical location on CPV.

**Fig 5:**
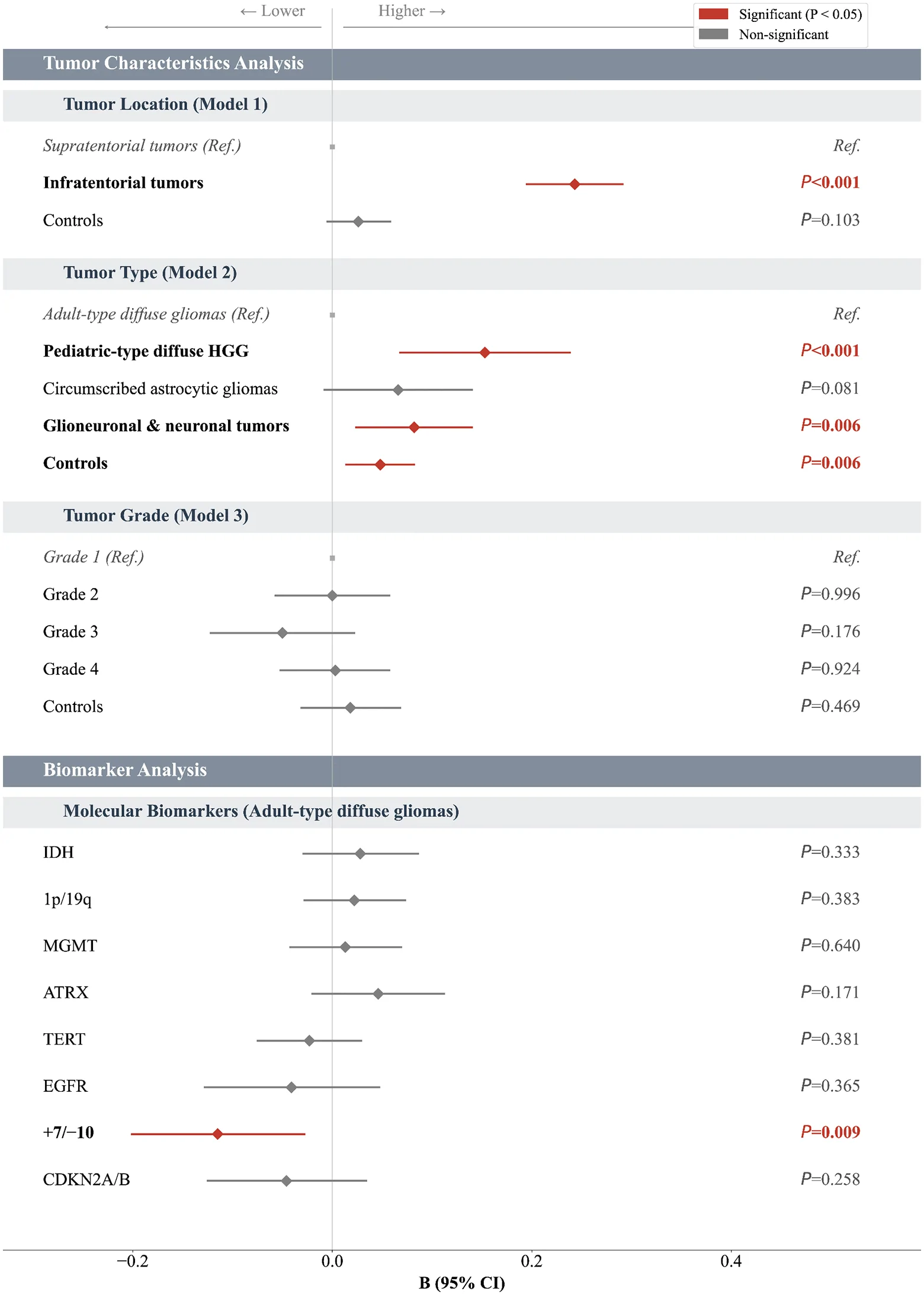
Forest plot of adjusted associations between tumor characteristics, molecular biomarkers, and CPV/TIV. Model 1 was adjusted for age, sex, and LVV. Model 2 and Model 3 were additionally adjusted for tumor location. Molecular biomarker analyses were adjusted for age, sex, and LVV. *B*, unstandardized coefficient; CI, confidence interval. Abbreviations: HGG, high-grade glioma; CPV, choroid plexus volume; TIV, total intracranial volume; LVV, lateral ventricular volume.

We first evaluated the associations between CPV and anatomical locations while adjusting for age, sex, and LVV. When tumors were classified into broad anatomical categories (supratentorial vs. infratentorial), regression analysis revealed that patients with infratentorial tumors showed significantly larger CPV compared with both controls and patients with supratentorial tumors (both *P* < 0.001), whereas CPV in patients with supratentorial tumors did not differ significantly from that in controls (Fig 5 and S7 Table in S1 File).

#### Regression analysis of the effect of tumor type on CPV.

To evaluate whether histological classification was associated with CPV independently of tumor location, tumor type and location were entered simultaneously into a multivariable linear regression model, adjusting for age, sex, and LVV.

Pairwise comparisons based on the adjusted model showed that patients with pediatric-type diffuse high-grade gliomas exhibited significantly larger CPV than all other categories except those with GNTs. Adult-type diffuse gliomas and circumscribed astrocytic gliomas showed comparable CPV. Controls demonstrated intermediate CPV values, differing significantly from adult-type diffuse and pediatric-type diffuse high-grade gliomas but not from circumscribed astrocytic gliomas or GNTs. Taken together, the adjusted results showed a general pattern of relatively lower CPV in adult-type diffuse gliomas and circumscribed astrocytic gliomas, intermediate values in controls, and relatively higher CPV in GNTs and pediatric-type diffuse high-grade gliomas (Fig 5 and S7 Table in S1 File).

To further explore potential subtype-specific effects, a separate regression model restricted to adult-type diffuse gliomas was conducted. After adjusting for age, sex, LVV, and location, no significant CPV differences were observed among patients with astrocytomas, oligodendrogliomas, and glioblastomas (all *P* > 0.05).

#### Regression analysis of the effect of tumor grade on CPV.

To assess whether histopathological grade was independently associated with CPV, we next modeled tumor grade together with location, adjusting for the same covariates.

Compared with controls, CPV differed modestly in patients with Grade 3 tumors (*P* = 0.027), whereas no significant differences were observed for Grades 1, 2, or 4. Importantly, among patients, there were no significant pairwise differences in CPV across Grades 1–4, indicating that histopathological grade was not independently associated with CPV after accounting for tumor location and other covariates (all *P* > 0.05).

Additional regression models incorporating scanner type as a covariate yielded materially unchanged results. Specifically, infratentorial location remained independently associated with increased CPV, tumor type showed comparable patterns of association, and tumor grade remained non-significant (Fig 5 and S7 Table in S1 File).

### Association of molecular biomarkers with CPV in adult-type diffuse gliomas

Given that adult-type diffuse gliomas constituted the largest subgroup, we further examined the associations between molecular biomarkers and CPV. The numbers of patients with available molecular data were 429 for IDH, 400 for 1p/19q, 399 for MGMT, 275 for ATRX, 388 for TERT, 223 for EGFR, 209 for +7/ − 10, and 218 for CDKN2A/B.

In exploratory molecular analyses, chromosome +7/ − 10 was associated with lower CPV after adjustment for age, sex, and LVV (*β* = –0.127, *P* = 0.009), and this association remained significant after further adjustment for scanner type (*β* = –0.131, *P* = 0.008) (Fig 5 and S8 Table in S1 File). No other molecular biomarkers—including IDH mutation, 1p/19q codeletion, MGMT promoter methylation, ATRX mutation, TERT promoter status, EGFR amplification, or CDKN2A/B deletion—were significantly associated with CPV in either model (all *P* > 0.05).

To further assess potential confounding, additional sensitivity analyses were performed for +7/ − 10 by separately adjusting for tumor location, WHO grade, tumor subtype, and IDH status. The association between +7/ − 10 and lower CPV remained statistically significant in all additional models (all P < 0.05; S9 Table in S1 File).

## Discussion

In this study, we examined patterns of CPV variation across gliomas and GNTs while considering demographic, ventricular, and anatomical factors. The findings suggest that CPV differences are closely linked to tumor location, particularly infratentorial involvement, whereas histopathological grade does not appear to exert an independent influence. In addition, certain tumor types showed distinct volumetric patterns beyond the effect of anatomical distribution. These observations provide insight into the associations between structural and biological tumor characteristics and ChP morphology.

### Age-related variations in CPV

CPV increased progressively with age in the control cohort, consistent with previous studies [22,23]. A similar trend was observed in adult patients, suggesting that age is positively associated with CPV.

In the elderly group, patients showed slightly smaller CPV compared with age-matched controls. Although this finding may suggest a potential difference between patients and controls in older individuals, the small magnitude of the difference and borderline statistical significance (*P* = 0.048) render the result inconclusive. This trend may reflect age-related alterations in brain structure or CSF dynamics but requires further confirmation. Conversely, pediatric patients exhibited markedly larger CPV than pediatric controls, and ventricular enlargement was also qualitatively observed in some pediatric patients. These findings highlight distinct age-related and disease-related patterns and underscore the importance of accounting for both age and ventricular volume when interpreting CPV alterations.

### Influence of anatomical location and tumor type on CPV

Anatomical location was strongly and consistently associated with CPV both before and after covariate adjustment. Infratentorial tumors were associated with markedly larger CPV compared with supratentorial tumors and controls, whereas the latter two groups showed comparable values. This enlargement may partly reflect alterations in CSF dynamics, including ventricular enlargement or obstructive hydrocephalus related to compression of the fourth ventricle or cerebral aqueduct [24,25]. However, the degree of ventricular obstruction was not directly quantified in the present study, and other mechanisms cannot be excluded. This interpretation may be particularly relevant in pediatric patients, whose tumors frequently arise in the posterior fossa or along the midline, where CSF pathway obstruction is common.

The type-related pattern observed in the multivariable models differed from that obtained by nonparametric analyses. In the unadjusted comparisons, pediatric-type diffuse and circumscribed astrocytic gliomas showed higher CPV than controls, adult-type diffuse gliomas, and GNTs. After adjustment for age, sex, LVV, and location, this pattern changed: pediatric-type diffuse high-grade gliomas and GNTs exhibited the largest CPV, whereas adult-type diffuse gliomas and circumscribed astrocytic gliomas showed relatively smaller values, indicating that anatomical distribution and ventricular volume contributed substantially to the differences observed in the unadjusted analyses.

After accounting for these covariates, pediatric-type diffuse high-grade gliomas, which include biologically aggressive diffuse midline gliomas [26,27], showed the most pronounced CPV enlargement. This finding may be compatible with several potential mechanisms, including altered CSF dynamics and tumor-associated inflammatory processes. However, the present imaging data do not allow these mechanisms to be distinguished, and the biological basis of the observed CPV differences remains uncertain.

Within adult-type diffuse gliomas, no significant CPV differences were observed among astrocytomas, oligodendrogliomas, and glioblastomas after adjusting for relevant covariates. This suggests that, despite molecular and clinical heterogeneity, these subtypes show broadly similar CPV profiles. The absence of distinct volumetric differences suggests that CPV alterations in adult-type diffuse gliomas may not be strongly determined by histological subtype alone and may instead reflect shared tumor-related factors, such as ventricular remodeling or other structural changes.

Notably, tumor grade itself was not independently associated with CPV once location and covariates were accounted for. This suggests that the apparent grade-related differences observed in unadjusted analyses primarily stem from differences in anatomical distribution rather than histopathological grade itself. Collectively, these results emphasize that anatomical and histological features, rather than tumor grade per se, are more strongly associated with ChP morphology across tumor types.

### Associations between CPV and molecular biomarkers

In the exploratory biomarker analysis of adult-type diffuse gliomas, most molecular biomarkers were not associated with CPV after adjustment for age, sex, and LVV. Specifically, IDH mutation, 1p/19q codeletion, MGMT promoter methylation, ATRX mutation, TERT promoter status, EGFR amplification, and CDKN2A/B deletion did not demonstrate significant associations.

Notably, + 7/ − 10 status was associated with lower CPV, and this association remained statistically significant in sensitivity analyses with separate adjustment for tumor location, WHO grade, tumor subtype, and IDH status. The + 7/ − 10 signature, characterized by combined chromosome 7 gain and chromosome 10 loss, is an important molecular feature of IDH-wildtype gliomas and is associated with aggressive tumor biology [28,29]. Mechanistically, it has been proposed that loss of tumor suppressor genes on chromosome 10 may be functionally compensated by oncogene activation on chromosome 7, conferring a clonal survival advantage [29]. However, the biological basis of its association with CPV remains unclear. Given the exploratory nature of the molecular analyses, this finding should be interpreted cautiously and requires validation in independent cohorts. Further radiogenomic and mechanistic studies are needed to determine whether and how tumor molecular characteristics are related to ChP morphology.

From a clinical perspective, automated CPV quantification may provide complementary information to conventional tumor-centered MRI assessment. Standardized MRI phenotyping frameworks, such as the updated VASARI 2.0 lexicon [30], have demonstrated the potential of conventional MRI features for glioma grading and IDH status prediction, highlighting the value of structured imaging characterization in neuro-oncology. The potential utility of CPV could include imaging-based characterization, prognostic stratification, and longitudinal treatment monitoring. However, the present cross-sectional study was not designed to evaluate diagnostic accuracy, clinical outcomes, or treatment response. Therefore, the clinical value of CPV remains to be established and requires prospective longitudinal validation before it can be considered for clinical implementation.

### Limitations and future directions

Several limitations should be acknowledged. First, MRI data were acquired using multiple scanners, which may introduce systematic variability despite standardized acquisition protocols. Although sensitivity analyses incorporating scanner type did not materially alter the observed associations, residual scanner-related effects cannot be entirely excluded. Second, some subgroups had relatively small sample sizes, which may reduce statistical power and contribute to instability in specific comparisons. Third, although LVV was included as a covariate to account for ventricular anatomy and the potential influence of ventricular enlargement or hydrocephalus, tumor volume, the degree of ventricular obstruction, and mass effect were not systematically quantified, and their residual effects on CPV cannot be entirely excluded. Patients with substantial tumor-related compression of the lateral ventricles or ChP that interfered with segmentation were excluded, but more subtle effects of tumor-related ventricular obstruction or mass effect may still have influenced CPV. In addition, this study focused on volumetric alterations of the ChP but did not assess its microstructural or functional characteristics, such as perfusion, permeability, or inflammatory marker expression. Finally, the retrospective design and the single-center source of the patient cohort may limit the generalizability of the findings.

Future research should incorporate multicenter datasets, larger sample sizes, and multimodal imaging approaches to validate and expand these findings. Moreover, CPV alone may not serve as a standalone biomarker but could be integrated with molecular, CSF, or functional imaging data to better elucidate the role of the ChP in the tumor microenvironment and brain homeostasis.

## Conclusion

This study characterizes CPV variation across gliomas and GNTs from anatomical and molecular perspectives. CPV differences were primarily associated with tumor location and histological classification, whereas tumor grade showed no independent association with CPV. An exploratory association between +7/ − 10 status and lower CPV was also observed, although this finding requires further validation. Overall, these findings suggest that CPV variation is associated with both anatomical and tumor-related characteristics. CPV warrants further investigation as a complementary imaging marker in prospective and longitudinal studies.

## Supporting information

S1 File Supporting information. (DOCX)

## Abbreviations

GNTs: glioneuronal and neuronal tumors
ChP: choroid plexus
CPV: choroid plexus volume
CSF: cerebrospinal fluid
CNS: central nervous system
TIV: total intracranial volume
LVV: lateral ventricular volume
