# Supplementary material for: Choroid plexus volume variation in patients with gliomas, glioneuronal and neuronal tumors: Associations with tumor characteristics
Source: PLoS One. 2026 Sep 15;21(9):e0357498. doi: 10.1371/journal.pone.0357498 (PMC13577507; doi:10.1371/journal.pone.0357498)
Supplement: Supplementary file 1 [file pone.0357498.s001.docx]

**Supporting information**

1. **Methods**
   1.1 **Choroid plexus volume measurement**

The choroid plexus (ChP) was segmented on 3D T1-weighted images using a deep learning algorithm developed by the authors based on the 3D nnU-Net framework.

The training dataset consisted of 50 glioma patients scanned on three 3.0 T MRI scanners [Siemens Prisma (n = 10), Philips Ingenia CX (n = 33), and Philips Ingenia (n = 7)]. The ChP was manually delineated by a neuroradiologist (J. Sun, with 6 years of experience in neuroradiology), and the segmentation results were subsequently reviewed and modified by another neuroradiologist (Y. Duan, with 17 years of experience in neuroradiology). All 50 annotated cases were used for model training.

The trained model was additionally evaluated in 30 cases that were not included in model training, including six cases from each of the five MRI scanner models. The validation cohort comprised 17 adult and 13 pediatric participants. The mean Dice similarity coefficient was 0.909 ± 0.044. The scanner-specific Dice coefficients were 0.919 ± 0.038 for Siemens Prisma, 0.909 ± 0.038 for Siemens Trio Tim, 0.897 ± 0.067 for GE Premier, 0.939 ± 0.014 for Philips Ingenia CX, and 0.882 ± 0.041 for Philips Ingenia. No significant differences in Dice performance were observed across scanner models (P = 0.188). The mean Dice coefficients were 0.914 ± 0.051 in adults and 0.903 ± 0.035 in pediatric participants, with no significant difference between the two age groups (P = 0.277).

The developed model was subsequently applied to all 3D T1-weighted images included in the study, and ChP volume (CPV) was calculated from the resulting segmentation masks.

1.2 **Intracranial volume measurement**

In this study, the total intracranial volume (TIV) was defined as the volume of all intracranial contents after excluding the skull, including brain parenchyma (gray and white matter), cerebrospinal fluid (CSF) primarily located in the ventricular system and subarachnoid space, and major intracranial vessels. The TIV does not include extracranial structures such as the scalp and eyeballs.

TIV was estimated using HD-BET, an advanced brain extraction algorithm based on artificial neural networks (ANNs). HD-BET was trained and validated on a large, multicenter MRI dataset obtained from 37 institutions across Europe. This dataset encompassed diverse MR scanners (e.g., GE, Philips, Siemens), multiple imaging sequences (T1WI, T2WI, FLAIR), and a wide range of brain conditions including tumors, trauma, and post-treatment changes. The model demonstrated high robustness and adaptability to various imaging conditions and pathological alterations.

The algorithm is based on a U-Net architecture, which is widely recognized for its superior performance in medical image segmentation tasks. Through multiple convolutional layers, HD-BET enables automatic identification of brain boundaries and accurate estimation of intracranial volume. During its development, five-fold cross-validation was used to ensure generalizability and stability of the model. Its high performance on independent test datasets further confirmed its robustness across different MRI platforms and acquisition protocols.

To ensure accuracy, all automatically segmented TIV outputs were reviewed by two trained neuroradiology researchers. This quality control process—combining automated segmentation with expert validation—enhanced the reliability of volumetric estimates and ensured a high-quality data foundation for subsequent neuroimaging analysis.

2. **Results**
   2.1 **Comparison of CPV across tumor types**

To further explore the differences in CPV within each tumor type, we performed 1:1 case-control matching separately between controls and patients across four tumor categories (Adult-type diffuse gliomas, Pediatric-type diffuse high-grade gliomas, Circumscribed astrocytic gliomas, and Glioneuronal and neuronal tumors). Detailed comparisons were then made between controls and each matched patient group.

First, we separately analyzed the CPV of patients across different subtypes and grades of adult-type diffuse gliomas. The results indicated no statistically significant differences among the three subtypes (astrocytoma, oligodendroglioma, and glioblastoma) or across the three tumor grades. However, patients with oligodendroglioma exhibited smaller CPV compared to controls (*P* = 0.016), while Grade 3 glioma patients had slightly smaller CPV than controls (*P* = 0.031) (S10 Table). We then expanded the analysis to all enrolled adult-type diffuse glioma patients beyond the matched cohort, focusing on CPV comparisons across grades within each tumor subtype. Within astrocytomas, no significant differences were observed among patients with Grades 2, 3, and 4 tumors (*P* = 0.970). Similarly, no significant differences were found between Grades 2 and 3 in oligodendrogliomas (*P* = 0.610) (S11 Table).

For GNTs, initial comparisons showed that GNT patients had larger CPV compared to controls (*P* = 0.023). This result differed from the previous findings (*P* = 1.000) where controls were compared with patients across all four tumor types using multiple comparisons. Further subdivision by tumor location revealed that patients with supratentorial GNTs and controls had similar CPV, both smaller than that in patients with infratentorial GNTs (*P* = 0.018, *P* = 0.001, respectively) (S12 Table).

When comparing the control group with diffuse midline glioma patients, CPV was significantly larger in diffuse midline glioma patients (*P* < 0.001) (S12 Table).

Finally, we compared controls with patients who have circumscribed astrocytic gliomas, specifically pilocytic astrocytoma and pleomorphic xanthoastrocytoma. The results indicated that CPV was similar in controls and patients with pleomorphic xanthoastrocytoma and was lower in both groups than in pilocytic astrocytoma patients (*P* < 0.001, *P* = 0.009, respectively) (S12 Table).

**S1 Table. Histological composition of glioneuronal and neuronal tumor cohort**

| Histological diagnosis | Numbers | Percentage |
| --- | --- | --- |
| Ganglioglioma | 70 | 51.1% |
| Glioneuronal tumor* | 36 | 26.3% |
| Dysembryoplastic neuroepithelial tumor | 9 | 6.6% |
| Rosette‐forming glioneuronal tumor | 8 | 5.8% |
| Papillary glioneuronal tumor | 7 | 5.1% |
| Multinodular and vacuolating neuronal tumor | 2 | 1.5% |
| Central neurocytoma | 2 | 1.5% |
| Diffuse glioneuronal tumor with oligodendroglioma‐like features and nuclear clusters | 1 | 0.7% |
| Diffuse leptomeningeal glioneuronal tumor | 1 | 0.7% |
| Extraventricular neurocytoma | 1 | 0.7% |

All diagnoses were confirmed according to the 5th edition of the WHO Classification of Tumors of the Central Nervous System.

*: Glioneuronal tumor refers to cases reported as mixed glioneuronal tumors in the original pathology reports without further WHO subclassification.

**S2 Table. MRI acquisition parameters for each scanner**

| Cohort | Scanner | N | 2D or 3D | FA (degree) | TR/TE (ms) | TI (ms) | Spatial Resolution (mm×mm×mm) | Matrix Size | Slices |
| --- | --- | --- | --- | --- | --- | --- | --- | --- | --- |
| Controls | Philips Ingenia CX | 542 | 3D | 8 | 7.0/3.0 | 880 | 1×1×1 | 256×256 | 196 |
|  | Siemens Trio Tim | 202 | 3D | 8 | 2600/3.0 | NA | 1×1×1 | 256×256 | 176 |
| Patients | Siemens Prisma | 119 | 3D | 8 | 1560/1.7 | 778 | 1×1×1 | 256×232, 256×256 | 192 |
|  | Philips Ingenia | 202 | 3D | 8 | 6.7-7.0 / 3.0-3.2 | NA | 1×1×1 | 220×218, 240×240 | 170-196 |
|  | Philips Ingenia CX | 449 | 3D | 8 | 6.4-11.2 / 3.0-5.1 | NA | 1×1×1 | 240×240 | 175 |
|  | GE Premier | 41 | 3D | 12 | 7.2-7.3 / 3.0 | 450 | 1×1×1 | 256×256 | 176 |

NA, not available

**S3 Table. Comparison of controls and patients before and after case-control matching**

|  | Before case-control matching | | | | |  | After case-control matching | | | |
| --- | --- | --- | --- | --- | --- | --- | --- | --- | --- | --- |
|  | Controls  (n=744) | Patients  (n=811) | | *Z/χ^2^* | *P* |  | Controls  (n=584) | Patients  (n=584) | *Z/χ^2^* | *P* |
| Age [years, median (IQR)] | 45 (16-54) | | 35 (17-49) | -6.03 | *P*<0.001 |  | 42 (15-52) | 42 (14-53) | -0.19 | *P*=0.849 |
| Sex |  | |  | 1.33^*^ | *P*=0.250 |  |  |  | 0.00^*^ | *P*=1.000 |
| Male [n (%)] | 407 (54.7%) | | 420 (51.8%) |  |  |  | 313 (53.6%) | 313 (53.6%) |  |  |
| Female [n (%)] | 337 (45.3%) | | 391 (48.2%) |  |  |  | 271 (46.4%) | 271 (46.4%) |  |  |
| CPV [mm^3^, median (IQR)] | 1625 (1300-1949) | | 1731 (1365-2157) | -4.93 | *P*<0.001 |  | 1610 (1286-1944) | 1764 (1400-2201) | -5.43 | *P*<0.001 |
| TIV [mm^3^, median (IQR)] | 1370660 (1283717-1453205) | | 1431301 (1339608-1521090) | -9.18 | *P*<0.001 |  | 1370890 (1281956-1452739) | 1431023 (1339471-1530765) | -8.08 | *P*<0.001 |
| CPV/TIV [×10^-3^, median (IQR)] | 1.19 (0.96-1.40) | | 1.21 (0.97-1.52) | -2.64 | *P*=0.008 |  | 1.17 (0.95-1.40) | 1.23 (1.00-1.53) | -3.52 | *P*<0.001 |

Values marked with * represent χ² values, others represent Z values.

Abbreviations: CPV, choroid plexus volume; TIV, total intracranial volume

**S4 Table. Comparison of CPV/TIV across different age groups after case-control matching**

| Group | Age (years) | Number (%) | CPV/TIV  [×10^-3^, median (IQR)] | Kruskal-Wallis H | |
| --- | --- | --- | --- | --- | --- |
|  |  |  |  | *H* | *P* |
| Controls  (n=584) | <18 | 172 (29.5%) | 0.93 (0.76-1.16) ^a^ | 133.22 | *P*<0.001 |
|  | 18-44 | 156 (26.7%) | 1.19 (0.97-1.39) ^b^ |  |  |
|  | 45-59 | 188 (32.2%) | 1.28 (1.09-1.47) ^c^ |  |  |
|  | ≥60 | 68 (11.6%) | 1.41 (1.22-1.69) ^d^ |  |  |
| Patients (n=584) | <18 | 163 (27.9%) | 1.27 (0.99-1.71) ^a^ | 13.42 | *P*=0.004 |
|  | 18-44 | 165 (28.3%) | 1.16 (0.91-1.35) ^b^ |  |  |
|  | 45-59 | 188 (32.2%) | 1.25 (1.04-1.52) ^ab^ |  |  |
|  | ≥60 | 68 (11.6%) | 1.28 (1.08-1.62) ^a^ |  |  |

Groups sharing a common letter in the CPV/TIV column do not significantly differ, groups with different letters show significant differences. Significance values have been adjusted using the Bonferroni correction for multiple tests. Abbreviations: CPV, choroid plexus volume; TIV, total intracranial volume

**S5 Table. Comparison of CPV/TIV between controls and patients within each age group after case-control matching**

| Age (years) | Group | Number (%) | CPV/TIV  [×10^-3^, median (IQR)] | Mann-Whitney U | |
| --- | --- | --- | --- | --- | --- |
|  |  |  |  | *Z* | *P* |
| <18 | Controls | 172 (51.3%) | 0.93 (0.76-1.16) | -7.73 | *P*<0.001 |
|  | Patients | 163 (48.7%) | 1.27 (0.99-1.71) |  |  |
| 18-44 | Controls | 156 (48.6%) | 1.19 (0.97-1.39) | -0.75 | *P*=0.456 |
|  | Patients | 165 (51.4%) | 1.16 (0.91-1.35) |  |  |
| 45-59 | Controls | 188 (50.0%) | 1.28 (1.09-1.47) | -0.38 | *P*=0.702 |
|  | Patients | 188 (50.0%) | 1.25 (1.04-1.52) |  |  |
| ≥60 | Controls | 68 (50.0%) | 1.41 (1.22-1.69) | -1.98 | *P*=0.048 |
|  | Patients | 68 (50.0%) | 1.28 (1.08-1.62) |  |  |

Abbreviations: CPV, choroid plexus volume; TIV, total intracranial volume

**S6 Table. Comparison of CPV/TIV across grades and locations after case-control**

| Grade | Location | Number (%) | CPV/TIV  [×10^-3^, median (IQR)] | Mann-Whitney U | |
| --- | --- | --- | --- | --- | --- |
|  |  |  |  | *Z* | *P* |
| 1 | Supratentorial | 76 (54.7%) | 1.11 (0.91-1.50) | -4.50 | *P*<0.001 |
|  | Infratentorial | 63 (45.3%) | 1.59 (1.24-1.91) |  |  |
| 2 | Supratentorial | 165 (93.2%) | 1.17 (0.99-1.38) | -2.10 | *P*=0.036 |
|  | Infratentorial | 12 (6.8%) | 1.41(1.19-1.55) |  |  |
| 3 | Supratentorial | 74 (96.1%) | 1.14 (0.92-1.41) | -1.24 | *P*=0.216 |
|  | Infratentorial | 3 (3.9%) | 1.41^*^ |  |  |
| 4 | Supratentorial | 97 (50.8%) | 1.17 (1.04-1.46) | -2.22 | *P*=0.026 |
|  | Infratentorial | 94 (49.2%) | 1.34 (1.07-1.64) |  |  |

**matching**

^*^: Only three patients with grade 3 infratentorial tumors were included, making it impossible to calculate the interquartile range. Abbreviations: CPV, choroid plexus volume; TIV, total intracranial volume

**S7 Table. Multivariable linear regression models assessing associations of anatomical location, tumor type, and tumor grade with CPV/TIV**

| **Variables** | **B (95% CI) *** | ***β**** | ***P**** | **B (95% CI) **** | ***β***** | ***P***** | **Model** |
| --- | --- | --- | --- | --- | --- | --- | --- |
| **Tumor location** |  |  |  |  |  |  | Model 1 |
| Supratentorial tumors (Ref) | – | – | – | – | – | – |  |
| Controls | 0.026 (–0.005 to 0.058) | 0.035 | 0.103 | 0.039 (−0.002 to 0.081) | 0.052 | 0.065 |  |
| Infratentorial tumors | **0.243 (0.195 to 0.291)** | **0.234** | **< 0.001** | **0.226 (0.175 to 0.277)** | **0.217** | **<0.001** |  |
| **Tumor type** |  |  |  |  |  |  | Model 2 |
| Adult-type diffuse gliomas (Ref) | – | – | – | – | – | – |  |
| Pediatric-type diffuse high-grade gliomas | **0.153 (0.068 to 0.238)** | **0.105** | **< 0.001** | **0.145 (0.057 to 0.233)** | **0.099** | **0.001** |  |
| Circumscribed astrocytic gliomas | 0.066 (–0.008 to 0.140) | 0.048 | 0.081 | 0.045 (−0.031 to 0.121) | 0.032 | 0.247 |  |
| Glioneuronal and neuronal tumors | **0.082 (0.024 to 0.140)** | **0.062** | **0.006** | **0.061 (0.002 to 0.121)** | **0.046** | **0.044** |  |
| Controls | **0.048 (0.014 to 0.082)** | **0.064** | **0.006** | **0.055 (0.012 to 0.098)** | **0.073** | **0.012** |  |
| **Tumor grade** |  |  |  |  |  |  | Model 3 |
| Grade 1 (Ref) | – | – | – | – | – | – |  |
| Grade 2 | 0.000 (–0.057 to 0.057) | 0.000 | 0.996 | 0.012 (−0.045 to 0.069) | 0.012 | 0.673 |  |
| Grade 3 | –0.050 (–0.122 to 0.022) | –0.033 | 0.176 | −0.022 (−0.095 to 0.051) | −0.014 | 0.551 |  |
| Grade 4 | 0.003 (–0.052 to 0.057) | 0.002 | 0.924 | 0.021 (−0.034 to 0.076) | 0.020 | 0.452 |  |
| Controls | 0.018 (–0.031 to 0.068) | 0.024 | 0.469 | 0.046 (−0.013 to 0.106) | 0.061 | 0.129 |  |

*Primary models were adjusted for age, sex, and LVV. Tumor type (Model 2) and tumor grade (Model 3) were additionally adjusted for tumor location. **Additionally adjusted for scanner type. Reference categories: male (for sex), supratentorial tumors (for location), adult-type diffuse gliomas (for tumor type), and Grade 1 (for tumor grade). *B*, unstandardized coefficients; CI, confidence interval; *β*, standardized coefficients. Significant *P* values (< 0.05) are shown in bold. Abbreviations: CPV, choroid plexus volume; TIV, total intracranial volume; LVV, lateral ventricular volume

**S8 Table. Multivariable linear regression analyses of CPV/TIV with molecular biomarkers in adult-type diffuse gliomas**

| Biomarkers | status | Number (%) | *B* (95% CI) * | *β* * | *P** | *B* (95% CI) ** | | *β*** | *P*** |
| --- | --- | --- | --- | --- | --- | --- | --- | --- | --- |
| IDH | 0 | 115 (26.8%) | 0.028 (-0.029 to 0.086) | 0.037 | 0.333 | 0.035 (−0.022 to 0.092) | | 0.045 | 0.231 |
|  | 1 | 314 (73.2%) |  |  |  |  | |  |  |
| 1p/19q | 0 | 244 (61.0%) | 0.022 (-0.028 to 0.073) | 0.032 | 0.383 | 0.034 (−0.017 to 0.085) | | 0.049 | 0.190 |
|  | 1 | 156 (39.0%) |  |  |  |  | |  |  |
| MGMT | 0 | 112 (28.1%) | 0.013 (-0.042 to 0.069) | 0.017 | 0.640 | 0.015 (−0.040 to 0.070) | | 0.019 | 0.601 |
|  | 1 | 287 (71.9%) |  |  |  |  | |  |  |
| ATRX | 0 | 204 (74.2%) | 0.046 (-0.020 to 0.112) | 0.063 | 0.171 | 0.038 (−0.026 to 0.103) | | 0.052 | 0.244 |
|  | 1 | 71 (25.8%) |  |  |  |  | |  |  |
| TERT | 0 | 182 (46.9%) | -0.023 (-0.075 to 0.029) | -0.034 | 0.381 | −0.014 (−0.066 to 0.037) | | −0.021 | 0.585 |
|  | 1 | 206 (53.1%) |  |  |  |  | |  |  |
| EGFR | 0 | 195 (87.4%) | -0.041 (-0.128 to 0.047) | -0.042 | 0.365 | −0.055 (−0.143 to 0.034) | | −0.056 | 0.224 |
|  | 1 | 28 (12.6%) |  |  |  |  | |  |  |
| +7/-10 | 0 | 179 (85.6%) | -0.115 (-0.201 to -0.028) | -0.127 | **0.009** | −0.119 (−0.206 to −0.031) | | −0.131 | **0.008** |
|  | 1 | 30 (14.4%) |  |  |  |  | |  |  |
| CDKN2A/B | 0 | 178 (81.7%) | -0.046 (-0.125 to 0.034) | -0.056 | 0.258 | −0.058 (−0.138 to 0.023) | | −0.070 | 0.159 |
|  | 1 | 40 (18.3%) |  |  |  | |  |  |  |

*Adjusted for age, sex and lateral ventricular volume. **Additionally adjusted for scanner type. 0 indicates absence (no mutation, co-deletion, methylation, amplification, or chromosomal abnormality). 1 indicates the presence of any of the above. *B*, unstandardized coefficients; CI, confidence interval; *β*, standardized coefficients. Significant *P* values (< 0.05) are shown in bold. Abbreviations: CPV, choroid plexus volume; TIV, total intracranial volume

**S9 Table. Sensitivity analyses of the association between +7/−10 status and CPV/TIV after additional adjustment for tumor characteristics in adult-type diffuse gliomas**

| **Additional adjustment** | **Primary model*** | | | **Scanner-adjusted model**** | | |
| --- | --- | --- | --- | --- | --- | --- |
|  | ***B* (95% CI)** | ***β*** | ***P*** | ***B* (95% CI)** | ***β*** | ***P*** |
| None (base model) | -0.115 (-0.201 to -0.028) | -0.127 | 0.009 | -0.119 (-0.206 to -0.031) | -0.131 | 0.008 |
| Tumor location | -0.112 (-0.198 to -0.025) | -0.123 | 0.012 | -0.116 (-0.203 to -0.028) | -0.128 | 0.010 |
| WHO grade | -0.145 (-0.249 to -0.041) | -0.160 | 0.007 | -0.146 (-0.251 to -0.041) | -0.161 | 0.007 |
| Tumor subtype | -0.174 (-0.284 to -0.065) | -0.193 | 0.002 | -0.174 (-0.285 to -0.064) | -0.193 | 0.002 |
| IDH status | -0.107 (-0.206 to -0.008) | -0.118 | 0.034 | -0.110 (-0.210 to -0.011) | -0.122 | 0.030 |

*Primary models were adjusted for age, sex, and lateral ventricular volume (LVV). **Scanner-adjusted models included the same covariates with additional adjustment for scanner type. Tumor location, WHO grade, tumor subtype, and IDH status were entered separately in the corresponding models; the base model included no additional tumor-related variable. All reported *B*, 95% CIs, *β*, and *P* values refer to the association between +7/−10 status and CPV/TIV.

All analyses were restricted to adult-type diffuse glioma patients with available +7/−10 results (n = 209). Tumor location was categorized as supratentorial or infratentorial; WHO grade as Grades 2–4; tumor subtype as astrocytoma, oligodendroglioma, or glioblastoma; and IDH status as wildtype or mutant. Only five tumors in this subset were infratentorial, all of which were +7/−10 absent; therefore, the location-adjusted analysis should be interpreted cautiously. *B*, unstandardized coefficient; CI, confidence interval; *β*, standardized coefficient; Abbreviations: CPV, choroid plexus volume; TIV, total intracranial volume

**S10 Table. Comparison of CPV/TIV between controls and adult-type diffuse gliomas after case-control matching**

|  | Number (%) | CPV/TIV  [×10^-3^, median (IQR)] | Kruskal-Wallis H | | |
| --- | --- | --- | --- | --- | --- |
|  |  |  | *H* | | *P* |
| Controls | 363 (100%) | 1.27 (1.05-1.46) ^a^ | 10.76 | *P*=0.013 | |
| Astrocytoma | 128 (35.3%) | 1.21 (0.93-1.40) ^ab^ |  |  |  |
| Oligodendroglioma | 145 (39.9%) | 1.14 (0.94-1.42) ^b^ |  |  |  |
| Glioblastoma | 90 (24.8%) | 1.16 (1.04-1.46) ^ab^ |  |  |  |
| Controls | 363 (100%) | 1.27 (1.05-1.46) ^a^ | 11.00 | *P*=0.012 | |
| Grade 2 | 179 (49.3%) | 1.19 (0.96-1.40) ^ab^ |  |  |  |
| Grade 3 | 84 (23.1%) | 1.14 (0.91-1.41) ^b^ |  |  |  |
| Grade 4 | 100 (27.5%) | 1.16 (1.04-1.45) ^ab^ |  |  |  |

Groups sharing a common letter in the CPV/TIV column do not significantly differ, groups with different letters show significant differences. Significance values have been adjusted using the Bonferroni correction for multiple tests. Abbreviations: CPV, choroid plexus volume; TIV, total intracranial volume

**S11 Table. Comparison of CPV/TIV across grades in adult-type diffuse gliomas**

|  | Number (%) | | CPV/TIV [×10^-3^, median (IQR)] | Rank Sum test | |
| --- | --- | --- | --- | --- | --- |
|  |  |  |  | *H/Z* | *P* |
| Astrocytoma | |  |  | 0.06 | *P*=0.970 |
| Grade 2 | | 121 (68.8%) | 1.18 (0.94-1.37) |  |  |
| Grade 3 | | 41 (23.3%) | 1.21 (0.90-1.34) |  |  |
| Grade 4 | | 14 (8.0%) | 1.17 (0.97-1.51) |  |  |
| Oligodendroglioma | |  |  | -0.51 | *P*=0.610 |
| Grade 2 | | 104 (64.2%) | 1.14 (0.91-1.34) |  |  |
| Grade 3 | | 58 (35.8%) | 1.05 (0.92-1.44) |  |  |

Abbreviations: CPV, choroid plexus volume; TIV, total intracranial volume

**S12 Table. Comparison of CPV/TIV between controls and patients with different tumor types after** **case-control matching**

| Classification | Number (%) | CPV/TIV  [×10^-3^, median (IQR)] | Rank sum test | |
| --- | --- | --- | --- | --- |
|  |  |  | *Z/H* | *P* |
| Controls | 119 (100%) | 1.08 (0.89-1.29) ^a^ | -2.27 | *P*=0.023 |
| GNTs | 119 (100%) | 1.16 (0.93-1.49) ^b^ |  |  |
| Controls | 119 (100%) | 1.08 (0.89-1.29) ^a^ | 12.70 | *P*=0.002 |
| Supratentorial GNTs | 93 (78.2%) | 1.10 (0.91-1.36) ^a^ |  |  |
| Infratentorial GNTs | 26 (21.8%) | 1.51 (1.11-1.79) ^b^ |  |  |
| Controls | 105 (100%) | 0.93 (0.73-1.25) ^a^ | -6.87 | *P*<0.001 |
| Diffuse midline glioma | 105 (100%) | 1.37 (1.06-1.74) ^b^ |  |  |
| Controls | 106 (100%) | 1.01 (0.79-1.26) ^a^ | 53.71 | *P*<0.001 |
| Pilocytic astrocytoma | 91 (85.8%) | 1.52 (1.17-1.86) ^b^ |  |  |
| Pleomorphic xanthoastrocytoma | 15 (14.2%) | 1.16 (0.99-1.31) ^a^ |  |  |

Groups sharing a common letter in the CPV/TIV column do not significantly differ, groups with different letters show significant differences. Significance values have been adjusted using the Bonferroni correction for multiple tests. Abbreviations: CPV, choroid plexus volume; TIV, total intracranial volume; GNTs, glioneuronal and neuronal tumors
